# Pelvic Floor Morbidity Following Vaginal Delivery versus Cesarean Delivery: Systematic Review and Meta-Analysis

**DOI:** 10.3390/jcm10081652

**Published:** 2021-04-13

**Authors:** Juan A. Barca, Coral Bravo, Maria P. Pintado-Recarte, Ángel Asúnsolo, Ignacio Cueto-Hernández, Javier Ruiz-Labarta, Julia Buján, Miguel A. Ortega, Juan A. De León-Luis

**Affiliations:** 1 Department of Public and Maternal and Child Health, School of Medicine, Complutense University of Madrid, 28040 Madrid, Spain; barcajuanantonio@gmail.com (J.A.B.); cbravoarribas@gmail.com (C.B.); ppintadorec@yahoo.es (M.P.P.-R.); ignaciocuetohernandez@gmail.com (I.C.-H.); javruila@hotmail.com (J.R.-L.); jaleon@ucm.es (J.A.D.L.-L.); 2 Department of Obstetrics and Gynecology, University Hospital Gregorio Marañón, 28009 Madrid, Spain; 3 Health Research Institute Gregorio Marañón, 28009 Madrid, Spain; 4 Department of Surgery, Medical and Social Sciences, Faculty of Medicine and Health Sciences, University of Alcalá, Alcalá de Henares, 28801 Madrid, Spain; angel.asunsolo@uah.es; 5 Ramón y Cajal Institute of Healthcare Research (IRYCIS), 28034 Madrid, Spain; mjulia.bujan@uah.es; 6 Department of Epidemiology & Biostatistics, Graduate School of Public Health and Health Policy, University of New York, New York, NY 10027, USA; 7 Department of Medicine and Medical Specialties, Faculty of Medicine and Health Sciences, University of Alcalá, Alcalá de Henares, 28801 Madrid, Spain

**Keywords:** pelvic floor morbidity, pelvic floor disorder, delivery

## Abstract

Objective: To compare pelvic floor disorders between vaginal delivery (VD) and cesarean delivery (CD). Methods: For this study, a PUBMED database search was used, utilizing a combination of relevant medical subjects’ headings (MeSH) terms, with the following keywords: “Pelvic floor disorders” or “Pelvic floor morbidity” and “Delivery”. Search limits were articles in English or Spanish, about women, published from December 2009 to December 2019. The STATA 16 package was used for meta-analysis and data heterogeneity assessment. Results: Thirteen studies meeting eligibility criteria were identified comprising 1,597,303 participants. Abstract: Pelvic floor morbidity prevalence was Urinary Incontinence (UI) 27.9% (5411 patients in 7 studies with reported cases), Pelvic Organ Prolapse (POP) 14.2% (6019 patients in 8 studies with reported cases), and Anal Incontinence (AI) 0.4% (1,589,740 patients in 5 studies with reported cases). Our meta-analyses revealed significantly higher rates of all three morbidities and overall morbidity in the VD versus CD group: UI OR = 2.17, 95% CI 1.64–2.87, *p* for heterogeneity ≤ 0.0001, *I*^2^ = 84%; POP OR = 3.28, 95% CI 1.91–5.63, *p* for heterogenicity ≤ 0.043, *I*^2^ = 63%; AI OR = 1.53, 95% CI 1.32–1.77; *p* for heterogeneity ≤ 0.291, *I*^2^ = 20%; and overall morbidity (OR = 2.17, 95% CI 1.64–2.87; *p* for heterogeneity ≤ 0.0001, *I*^2^ = 84%). Conclusion: Vaginal delivery is directly related to the appearance of pelvic floor disorders, mainly UI, POP, and AI. The risk of POP should be taken into higher consideration after vaginal delivery and postpartum follow-up should be performed, to identify and/or treat it at the earliest stages.

## 1. Introduction

Pelvic floor disorders (PFD) affect millions of women worldwide after pregnancy and childbirth [1,2,3,4,5,6]. The main risk factors associated with PFD described in the literature are: age, overweight, primiparity/multiparity, chronic constipation, as well as method of delivery [5].

Introduction: In low-middle income countries, pregnancy and delivery are an important cause of maternal mortality [1], while in more developed countries they still account for a significant proportion of maternal morbidities postpartum. In the United States, 10% of women will need surgery for a PFD. Annual rates for urinary incontinence (UI) and pelvic organ prolapse (POP) surgery have been estimated to be 135,000 and 200,000 women, respectively. The prevalence of female UI was reported as 20–50%, and anal incontinence (AI) as 11–15% [2]. During pregnancy and childbirth, stress UI is the most common type, with an estimated prevalence during pregnancy of 31% [3].

Obstetric trauma, to which not only the pelvic floor musculature is subjected, but also all the structures of the urogenital sphere, can injure different anatomical areas, such as the vagina, perineum, deep pelvic floor musculature, sphincter, and even innervations at this level [4].

Ashton Miller and Delancey found approximately 10% of women will experience some type of pelvic floor disorder which may require surgery, especially related to vaginal delivery [7]. Furthermore, several studies have highlighted the importance and incidence of PFD in the female population, with an incidence of 33–40% of perineal injuries after childbirth. In fact, pelvic floor trauma caused by vaginal delivery is possibly underestimated when it reaches 33–40% of women without any perineal trauma prior to childbirth [8].

This systematic review was designed to examine the relationship between vaginal versus caesarean delivery and the subsequent development of PFD in women. In a recent study, vaginal vs. caesarean delivery, increases the risk 3 times of having UI. [9].

For this systematic review, we studied the most common pelvic floor disorders, as defined by scientific evidence, such as UI, AI, POP, and sexual disorders after childbirth. [10].

The aim of this review and meta-analysis, following the PICOS question model, is to study patients attended at the time of delivery, differentiating between vaginal and caesarean delivery, in order to compare these two groups, studying the differences between the incidences of pelvic floor disorders, and to determine to what extent vaginal delivery may have a higher incidence of different types of pelvic floor disorders.

## 2. Materials and Methods

### 2.1. Protocol, Eligibility Criteria, Information Sources, and Search Strategies

This study was registered within the PROSPERO database (registration number: CRD42019132674). This review was performed according to a-priori-designed protocol recommended for systematic reviews following PRISMA guidelines [11].

The following search criteria were followed in the revision, combining free text and associates terms MeSH from PUBMED database: 2009/12/01:2019/12/31[Date—Publication] AND ((„deliveries”[All Fields] OR „delivery, obstetric”[MeSH Terms] OR („delivery”[All Fields] AND „obstetric”[All Fields]) OR „obstetric delivery”[All Fields] OR „delivery”[All Fields]) AND („pelvic floor disorders”[MeSH Terms] OR ((„pelvic”[All Fields] AND „floor”[All Fields]) AND „disorders”[All Fields]) OR „pelvic floor disorders”[All Fields] OR ((„pelvic”[All Fields] AND „floor”[All Fields]) AND „diseases”[All Fields]) OR „pelvic floor diseases”[All Fields] OR ((„pelvic floor”[MeSH Terms] OR („pelvic”[All Fields] AND „floor”[All Fields]) OR „pelvic floor”[All Fields]) AND („epidemiology”[MeSH Subheading] OR „epidemiology”[All Fields] OR „morbidity”[All Fields] OR „morbidity”[MeSH Terms] OR „morbid”[All Fields] OR „morbidities”[All Fields] OR „morbids”[All Fields])))).

Search limits were research articles in English or Spanish, about women, published from December 2009 to December 2019. Exclusion criteria were reports of less than 50 patients, articles not reporting on delivery route, duplicate articles, those with no full-text available, or those describing studies outside an obstetrics context.

Reference lists of relevant articles were hand-searched for additional reports. A reference database (Endnote X7 Thompson Reuters) was used to incorporate all references.

### 2.2. Study Selection, Data Collection and Data Items

The primary outcomes explored in the present systematic review were: Pelvic floor disorders (PFD) defined by the scientific literature as urinary incontinence, fecal/anal incontinence, pelvic organ prolapse, sexual dysfunction, and chronic pelvic pain [10]. International Continence Society (ICS) defines urinary incontinence (UI) as involuntary leakage of urine due to exertion or exercise or secondary to sneezing or coughing, by sensation of urgency or mixed; anal incontinence (AI) as involuntary loss of solid or liquid feces or mucus; and pelvic organ prolapse (POP) as the descent of one or more of the anterior vaginal wall, posterior vaginal wall, uterus (cervix), or the apex of the vagina [12,13,14,15]. The presence of any such sign should be correlated with symptoms consistent with prolapse.

Other variables recorded were: author, publication year, period of study, country, type of study, type of questionnaire, number of participants in the study (N), mean maternal age (years), number, and percentage of vaginal deliveries (VD), number and percentage of eutocic or dystocic instrumental VD, number and percentage of Cesarean delivery (CD), number of UI episodes, number of AI episodes, and number of POP episodes.

Eutocic VD are procedures in which there is no instrumental usage. Instrumental deliveries are procedures using any instrument such as forceps, a vacuum, or a spatula.

Two authors reviewed all titles and abstracts independently. If the title and abstract did not offer enough information, the full text was retrieved. Potentially eligible studies were obtained and further evaluated. Final studies included in the review were selected by both authors after applying eligibility criteria independently. Disagreement between both researchers was resolved by consensus. Each reviewer collected the data independently and included them in a data extraction sheet with the studied variables. Discrepancies were resolved by both authors checking each study against the data sheet.

### 2.3. Risk of Bias and Statistical Analysis

Risk of bias was assessed independently by both authors by determining the adequacy of compliance with inclusion criteria. Items assessed were: consecutive recruitment, correct description of cases included, procedures undertaken, and complete reporting of outcomes and complications.

We tried to choose strict eligibility criteria to identify a good number of studies that were as homogeneous as possible, and thereby extract specific and valid conclusions. For example, to eliminate positive outcome bias, a uniform definition of PFD was used (UI, AI and POP) as the most frequent pathology in the female population [12,13,14,15]. Any disagreement was resolved by discussion.

The quality of the evidence of studies included was assessed according to the Grade of Evidence Working Group Criteria [16].

Calculation of OR between the different studies were carried out using the software STATA, version 16. Results were expressed as rates (%) for dichotomous variables and 95% confidence intervals (95% CI) calculated. For publication bias analysis Egger test and Rosenthal model were carried out, with *I*^2^ ≤ 70 for statistical significance. 

## 3. Results

**Figure 1:**
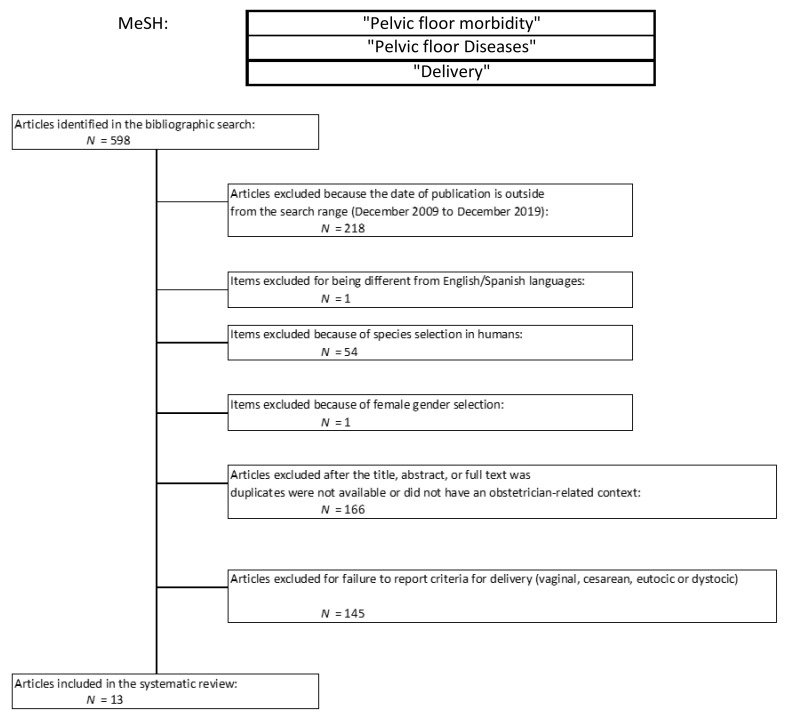
Study Flowchart.

The initial search retrieved 598 articles, of which only 13 were included in the meta-analysis after applying the exclusion criteria. Figure 1 shows the flowchart for article selection.

Figure 1 shows the flowchart of MeSH, with search limits and exclusion criteria, reflecting both the search-exclusion reasons and number of papers excluded in each criterion. Of 598 articles identified in the search, 13 articles met our established inclusion criteria.

### 3.1. Characteristic of the Included Studies

Table 1 shows the distribution of each of the variables collected from the studies. Nine of the 13 studies were published in the last 5 years (69.23%). The total number of participants was 1,597,303. All studies were in English language. The study by Larsson et al. was the main contributor including 1,586,154 patients [17].

### 3.2. Quality Assessment

Table 2 shows the heterogeneity of the studies selected. The results of the POP studies (*I*^2^ = 63.1%) were 6 times the ratio of heterogenicity to UI and tripled the ratio to AI. The results of UI were the most homogeneous among the studies selected for review

### 3.3. Results of the Systematic Review

**Figure 2:**
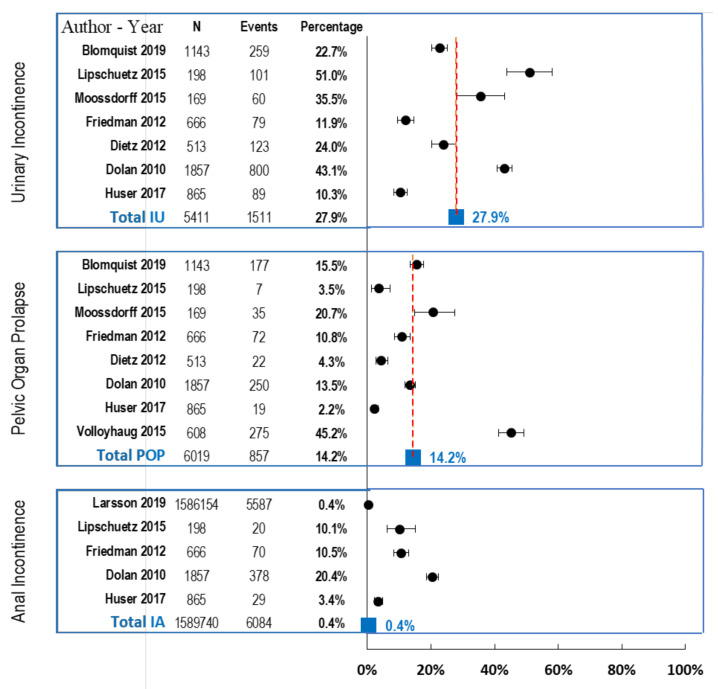
Event case study for Urinary Incontinence (UI), Pelvic Organ Prolapse (POP), Anal Incontinence (AI). OBS = *N* patients in each study, CI Confidence Interval and Forest plot.

Regarding morbidity events for each pathology, as shown in Figure 2, the prevalence of UI was the highest of all (27.9%, 95% CI 26.7–29.2), followed by POP at half the rate (14.2%, 95% CI 13.4–15.5), and finally AI with a very low prevalence (0.4%, CI 95% 0.4–0.4), with a high risk of bias, due to Larsson’s study [17], which had a larger *N* compared with the other studies.

In the study of events, 53.8% of the studies report UI, of which 42.8% had a UI rate above the average defined in the studies. The mean UI rate in study group with *N* = 5411 patients was 27.9%.

In the case of POP, 61.5% of the studies reported this disease, while 62.5% had a POP rate under the average of the review. Two studies with the highest *N* rates, those by Blomquist et al. [18] and Dolan et al. [29], were very close to the average prevalence of the study (14.2%), while smaller studies such as the studies of Lipscuetz et al. [25] and Volloyhaug et al. [26] were the furthest from the calculated average. The mean POP in the study group for an *N* = 6019 patients was 14.2%.

It should be noted that in the review only 38.5% of the studies reported AI, of which Larsson et al. [17] had the highest *N* and, therefore, defined the mean as 0.4% with an *N* = 1,589,740 patients, of whom only 0.2% were not included in the above-mentioned study. Therefore, we established that the AI ratio had a bias that did not allow subsequent conclusions to be drawn from the results obtained.

### 3.4. Results of the Meta-Analysis

#### 3.4.1. Meta-Analysis of Morbidity

**Figure 3:**
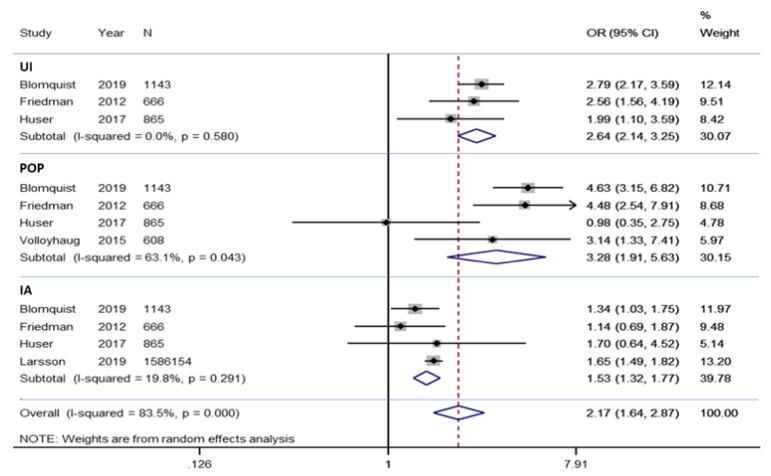
Forest plot for UI, POP, AI, overall in the Cesarean delivery and Vaginal delivery groups. OR, Odds Ratio.

There were five studies included in the meta-analysis of total PFD including UI, POP, and AI, with the 1,588,828 patients studied shown in Figure 3. The overall results reveal a higher prevalence of this morbidity in patients who had a VD versus a CD (OR = 2.17, 95% CI 1.64–2.87; *p* for heterogeneity ≤ 0.0001, *I*^2^ = 84%).

#### 3.4.2. Meta-Analysis of Urinary Incontinence

**Figure 4:**
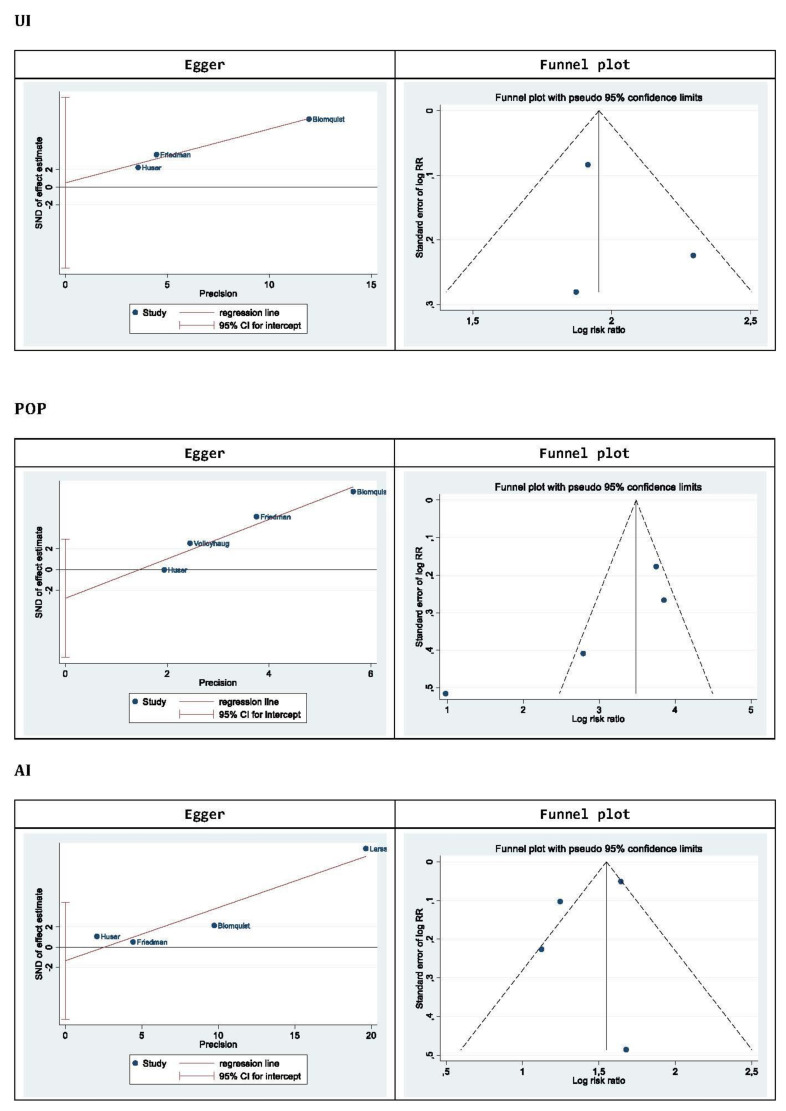
Publication risk of bias for meta-analysis results.

There were three studies included in the meta-analysis of UI morbidity, with the 2674 patients studied shown in Figure 3. The results reveal a higher prevalence of this morbidity in patients who had a VD versus a CD (OR = 2.64, 95% CI 2.14–3.25; *p* for heterogenicity ≤ 0.580, *I*^2^ = 0%). To evaluate the publication bias, two tests have been carried out, the Egger test and the funnel plot in Figure 4, by pathology. In the case of UI, the results do not offer a publication bias [annex I].

#### 3.4.3. Meta-Analysis of Pelvic Organ Prolapse

There were four studies included in the meta-analysis of POP morbidity, with the 3282 patients studied shown in Figure 3. The results reveal a higher prevalence of this morbidity in patients who had a VD versus a CD (OR = 3.28, 95% CI 1.91–5.63; *p* for heterogeneity 0.043, *I*^2^ ≤ 63%). In Figure 4, POP results do not offer a publication bias [annex I].

#### 3.4.4. Meta-Analysis of Anal Incontinence

There were four studies included in the meta-analysis of AI morbidity, with the 1,588,828 patients studied shown in Figure 3. The results reveal a higher prevalence of this morbidity in patients who had a VD versus a CD (OR = 1.53, 95% CI 1.32–1.77; *p* for heterogeneity ≤ 0.291, *I*^2^ = 20%). In Figure 4, AI results do not offer a publication bias [annex I].

### 3.5. Sensitivity Analysis

Meta-analysis showed a heterogeneity of overall morbidity higher (*I*^2^ = 84%) than recommended for a meta-analysis (*I*^2^ = ≈70%), due to different aspects of recruitment and methodology among the different studies. As overall morbidity *I*^2^ is >70% and no further publications bias was analyzed, we cannot consider the results.

We continued to carry out these tests because in the morbidities studied independently, the heterogeneity tests and publication bias tests are far below the acceptable limit, so we understand that the results are useful for the analysis.

However, this meta-analysis should be followed up by further studies that can address the deficiencies and limitations that have been identified in the heterogeneity of the included studies.

## 4. Discussion

This systematic review of the literature identified thirteen studies of interest involving over 1.5 million patients in the last ten years and representing ten countries. To our knowledge, this is the most up to date review of PFD of obstetric origin, analyzing the three most prevalent pelvic floor morbidities.

As shown in Table 1, in recent years, there has been a growing interest in studying the association between type of delivery and the risk of different pelvic floor disorders. Hage–Fransen et al. show that these risk factors are multifactorial and differ between the different pelvic floor disorders. Further research, along with long-term follow-up, is needed [30] to form solid conclusions. Pelvic tissue damage can generate UI, AI, and POP in the postpartum period or during other points in a woman’s life [31,32]. Major impact on women can occur from UI, POP, and AI which are diseases whose major etiology is the stress of childbirth and the form of delivery type.

The results obtained in this review indicate a UI prevalence in non-nulliparous women at 27.9%, which indicates that this problem needs to be considered to improve the quality of women’s lives once they have had their first child. Prevalences of female UI after delivery vary in the literature, where there is no consensus on what percentage is the most accurate, ranging from 15%–50%, depending on the study [31,32,33,34,35].

The data obtained in the review indicate a POP prevalence of 14.2% in non-nulliparous women. Reporting of POP is much more complex because a medical diagnosis is required after a vaginal exam, and in most studies, there are large biases, mainly because patients do not know how to tell if they have POP.

In this review, the prevalence of AI in relation to childbirth was 0.4% in non-nulliparous women, considering the bias produced by the inclusion of the study by Larsson et al., which included an N of more than 1,500,000 patients. AI occurs at a different prevalence rates in relation to the type of study and its inclusion methods. Therefore, we can find prevalence between 0.4–20% in the population [36,37]. Due to the large bias produced by the inclusion of the Larsson’s study, we cannot draw a conclusion from the data obtained in the review.

In this study, as shown in Figure 2, VD appears to double the prevalence of pelvic floor disorders compared with CD, namely more than twice the risk of UI, three times the risk of POP, and slightly less than two times the risk of AI (with the available data).

There are also several studies showing that elective CD can reduce the incidence of these pelvic floor pathologies such as incontinence. Although in the studies reviewed here, no distinction is made between elective and non-elective CS [38,39].

However, it should be noted that the majority of studies (62%) included in this systematic review and meta-analysis, were diagnosed PFD via questionnaires, while the smallest number of publications (38%) had a clinical diagnosis with ultrasound, medical diagnosis or pelvineometer as shown in Table 1.

As a result of the meta-analysis, we obtained acceptable ratios of heterogeneity. We can, therefore, conclude that the type of delivery has a direct impact on pelvic floor morbidity. Regarding the results of this study, vaginal delivery has significantly greater complication and morbidity effects than cesarean delivery. Some limitations should be avoided in future studies. Morbidity events by type of delivery (vaginal delivery/instrumental vaginal delivery) should be known and related within the vaginal delivery. More articles should be included in the analysis that meet the inclusion criteria.

The main factor we identified that affects the heterogenicity of the study was the lack of a homogeneous collection and presentation process of the methods and obstetric information of the patients included in the different studies. For future research, our recommendation would be to clearly define generic (VD/CD) and specific (vaginal delivery/instrumental vaginal delivery), Elective/Non-Elective, and obstetric variables to identify conclusions more precisely on the direct risks associated with each form of delivery.

Hage-Fransen et al., in their study, found UI during pregnancy, episiotomy, instrumental vaginal delivery tears, and constipation as risks factors for UI at postpartum. AI during pregnancy, maternal age > 35 years, prenatal BMI > 30 kg/m^2^, instrumental vaginal delivery, spontaneous vaginal delivery, oxytocin augmentation, and newborn > 4000 g as risk factors for AI at postpartum [30].

No clear association has been established between type of delivery and perineal dysfunction, while the preventive effect of cesarean delivery is highly controversial. The results of the published studies are questionable because they are retrospective and cohort studies. Randomized trials should be conducted by type of delivery (vaginal/caesarean) to have a clear view of comparison between types of delivery and perineal dysfunction.

The strength of this study is, this review and meta-analysis include 1,597,303 patients, from 10 different countries. However, there are different limitations in the study, that once identified, will be interesting to consider in future research. With the information from the studies included in this review and meta-analysis, it is not possible to determine which procedure led to a cesarean delivery, whether it was elective or emergency. Therefore, it has not been possible to perform a stratified analysis that could provide data on the causes of morbidity associated with each type of delivery. Similarly, there is no information in the studies included in this review on the morbidity events produced in each of the types of delivery, and they only provide information on the total events found in the global sample of the study. This prevents us from performing a meta-analysis by subgroups of instrumental vaginal delivery with variables such as BMI and maternal age, since we do not have the number of events (IU, POP, IA) among exposed and unexposed patients, so it is impossible to collect disaggregated event information to do so. Even though we contacted all corresponding authors by email, asking for these data in order to carry out the association study, unfortunately, only two of the teams replied, one indicating that they did not have the information available as it was a 2010 study. The other team gave us the required information, although the information with data from a single study was not relevant to perform a meta-analysis by subgroups of instrumental vaginal delivery.

## 5. Conclusions

The results of this study show that vaginal delivery is directly related to the appearance of pelvic floor pathologies, mainly IU, POP, and IA. Although the incidence of postpartum IU is higher than the others (27.9%), in vaginal delivery, however, the POP presents a higher risk OR = 3.28, compared to IU in cesarean deliveries.

Based on the results obtained in the study, it is necessary to consider the risk of POP after vaginal births, and a greater control of the possible appearance of POP during the postpartum period should be carried out in order to minimize and/or treat the early stages when it becomes evident that the patient could be affected.

Further studies are necessary, preferably randomized trials, to have a better understand of PFD knowledge. It would be interesting for future studies to include multi-factorial determinants identifying the type of vaginal delivery, with and without instrumentalization, and which cases the events of pelvic floor morbidity (UI, POP, AI) have occurred, in order to be able to relate each type of vaginal delivery to the pathology associated with that procedure. Knowledge of this topic helps physicians find effective strategies to reduce the likelihood of developing POPs after childbirth and to be able to pay more attention to this condition in postpartum pelvic floor care.

## Figures and Tables

**Table 1:** Characteristics of the included studies.

Author	Year	Period of Study	Country	Type of Study	Type of Questionnaire	No. of Patients	Mean Age (SD or Range)
Blomquist [18]	2019	October 2008–December 2013	USA	Longitudinal study	Prolapse-Incontinence Questionnaire	1143	40 (36.6 43.7)
Larsson [17]	2019	1973–2015	Sweden	Observational study	ICD8-10 diagnosis	1586.154	26.73 (5.57)
Fairchild [19]	2019	August 2014–July 2016	USA	Prospective cohort	POP-Q, Ultrasound	112	30.62 (N/A)
Colla [20]	2018	August 2016–May 2017	Brazil	Prospective observational	ICIQ_SF, POP-Q, PFM perineometer data	227	27 (26.03–27.68)
Abdool [21]	2017	November 2015–June 2016	South Africa	Observational study	ICIQ-VS Questionnaire	153	25.35 (18–38)
Huser [22]	2017	January 2002–December 2007	Czech	Prospective cohort	Internet-based survey	865	N/A
Yohay [23]	2016	March–July 2014	Israel	Prospective longitudinal cohort	PFDI-20 Questionnaire	117	30.84 (5.05)
Moossdorff [24]	2015	January 2010–December 2010	Netherlands	Observational study	Web based survey	169	29.74 (N/A)
Lipschuetz [25]	2015	N/A	Israel	Cross sectional study	PFBQ Questionnaire	198	28 (5.7)
Volloyhaug [26]	2015	January 1990–December 1997	Norway	Cross sectional study	POP-Q questionnaire, ultrasound	608	28.5 (N/A)
Friedman [27]	2012	N/A	USA	Prospective cohort	POP Quantification system	666	32.40 (N/A)
Dietz [28]	2012	October 2005–March 2010	Australia	Prospective cohort	Questions interview & ultrasound	513	27.5 (17–45)
Dolan [29]	2010	January 1983–August 1986	UK	Prospective cohort	Sheffield pelvic floor questionnaires	1857	26.2 (4.8)
Total (N)					1597.303		

N/A: not available.

**Table 2:** Heterogenicity test of the included studies.

	Heterogeneity Statistic	Degrees of Freedom	*p*	I-Squared **	Tau-Squared
UI	1.09	2	0.580	0.0%	0.000
POP	8.13	3	0.043	63.1%	0.1812
AI	3.74	3	0.291	19.8%	0.0058
Overall	60.46	10	0.000	83.5%	0.1535
	**z**	***p***			
UI	9.10	0.000			
POP	4.31	0.000			
AI	5.64	0.000			
Overall	5.40	0.000			

Significance test(s) of OR = 1. ** I-squared: the variation in OR attributable to heterogeneity.

## Data Availability

The data used to support the findings of the present study are available from the corresponding author upon request.

## Supplementary Materials

The following are available online at https://www.mdpi.com/article/10.3390/jcm10081652/s1, Annex I of work and search dynamics.

## Abbreviations

VD	Vaginal delivery
CD	Cesarean delivery
UI	Urinary incontinence
POP	Pelvic organ prolapse
AI	Anal incontinence
OR	Odds Ratio
CI	Confidence Interval
*I* ^2^	I squared
PFD	Pelvic floor disorders

## References

[1] Lan C.W., Tavrow P. (2017). Composite measures of women’s empowerment and their association with maternal mortality in low-income countries. BMC Pregnancy Childbirth.

[2] Kenton K., Mueller E.R. (2006). The global burden of female pelvic floor disorders. BJU Int..

[3] Martin-Martin S., Pascual-Fernandez A., Alvarez-Colomo C., Calvo-Gonzalez R., Muñoz-Moreno M., Cortiñas-Gonzalez J.R. (2014). Urinary incontinence during pregnancy and postpartum. Associated risk factors and influence of pelvic floor exercises. Arch. Esp. Urol..

[4] Rortveit G., Hannestad Y.S. (2014). Association between mode of delivery and pelvic floor dysfunction. Tidsskr. Nor. Legeforening.

[5] Wu J.M., Vaughan C.P., Goode P.S., Redden D.T., Burgio K.L., Richter H.E., Markland A.D. (2014). Prevalence and trends of symptomatic pelvic floor disorders in U.S. women. Obstet. Gynecol..

[6] Maclennan A.H., Taylor A.W., Wilson D.H., Wilson D. (2000). The prevalence of pelvic floor disorders and their relationship to gender, age, parity and mode of delivery. BJOG.

[7] Ashton-Miller J.A., Delancey J.O. (2009). On the biomechanics of vaginal birth and common sequelae. Annu. Rev. Biomed. Eng..

[8] Caudwell-Hall J., Atan I.K., Rojas R.G., Langer S., Shek K.L., Dietz H.P. (2018). Atraumatic normal vaginal delivery: How many women get what they want?. Am. J. Obstet. Gynecol..

[9] Siahkal S.F., Iravani M., Mohaghegh Z., Sharifipour F., Zahedian M. (2020). Maternal, obstetrical and neonatal risk factors’ impact on female urinary incontinence: A systematic review. Int. Urogynecol. J..

[10] Bo K., Frawley H.C., Haylen B.T., Abramov Y., Almeida F.G., Berghmans B., Bortolini M., Dumoulin C., Gomes M., McClurg D. (2017). An International Urogynecological Association (IUGA)/International Continence Society (ICS) joint report on the terminology for the conservative and nonpharmacological management of female pelvic floor dysfunction. Int. Urogynecol. J..

[11] Liberati A., Altman D.G., Tetzlaff J., Mulrow C., Gøtzsche P.C., Ioannidis J.P.A., Clarke M., Devereaux P.J., Kleijnen J., Moher D. (2009). The Prisma statement for reporting systematic reviews and meta-analyses of studies that evaluate health care interventions: Explanation and elaboration. J. Clin. Epidemiol..

[12] Abrams P., Cardozo L., Fall M., Griffiths D., Rosier P., Ulmsten U., Van Kerrebroeck P., Victor A., Wein A. (2002). The standardisation of terminology in lower urinary tract function: Report from the standardisation sub-committee of the International Continence Society. Neurourol. Urodyn..

[13] Whitehead W.E., Borrud L., Goode P.S., Meikle S., Mueller E.R., Tuteja A., Weidner A., Weinstein M., Ye W., Pelvic Floor Disorders Network (2009). Fecal incontinence in US adults: Epidemiology and risk factors. Gastroenterology.

[14] Iglesia C.B., Smithling K.R. (2017). Pelvic Organ Prolapse. Am. Fam. Physician.

[15] Dietz H.P., Lanzarone V. (2005). Levator trauma after vaginal delivery. Obstet. Gynecol..

[16] Guyatt G.H., Oxman A.D., Vist G.E., Kunz R., Falck-Ytter Y., Alonso-Coello P., Schünemann H.J. (2008). GRADE: An emerging consensus on rating quality of evidence and strength of recommendations. BMJ.

[17] Larsson C., Hedberg C.L., Lundgren E., Söderström L., Tunón K., Nordin P. (2019). Anal incontinence after caesarean and vaginal delivery in Sweden: A national population-based study. Lancet.

[18] Blomquist J.L., Carroll M., Muñoz A., Handa V.L. (2020). Pelvic floor muscle strength and the incidence of pelvic floor disorders after vaginal and cesarean delivery. Am. J. Obstet. Gynecol..

[19] Fairchild P.S., Low L.K., Kowalk K.M., Kolenic G.E., DeLancey J.O., Fenner D.E. (2019). Defining “normal recovery” of pelvic floor function and appearance in a high-risk vaginal delivery cohort. Int. Urogynecol. J..

[20] Colla C., Paiva L.L., Ferla L., Trento M.J.B., De Vargas I.M.P., Dos Santos B.A., Ferreira C.F., Ramos J.G.L. (2018). Pelvic floor dysfunction in the immediate puerperium, and 1 and 3 months after vaginal or cesarean delivery. Int. J. Gynaecol. Obstet..

[21] Abdool Z., Lindeque B.G., Dietz H.P. (2018). The impact of childbirth on pelvic floor morphology in primiparous Black South African women: A prospective longitudinal observational study. Int. Urogynecol. J..

[22] Huser M., Janku P., Hudecek R., Zbozinkova Z., Bursa M., Unzeitig V., Ventruba P. (2017). Pelvic floor dysfunction after vaginal and cesarean delivery among singleton primiparas. Int. J. Gynaecol. Obstet..

[23] Yohay D., Weintraub A.Y., Mauer-Perry N., Peri C., Kafri R., Yohay Z., Bashiri A. (2016). Prevalence and trends of pelvic floor disorders in late pregnancy and after delivery in a cohort of Israeli women using the PFDI-20. Eur. J. Obstet. Gynecol. Reprod. Biol..

[24] Moossdorff-Steinhauser H.F.A., Albers-Heitner P., Weemhoff M., Spaanderman M.E.A., Nieman F.H.M., Berghmansa B. (2015). Factors influencing postpartum women’s willingness to participate in a preventive pelvic floor muscle training program: A web-based survey. Eur. J. Obstet. Gynecol. Reprod. Biol..

[25] Lipschuetz M., Cohen S.M., Liebergall-Wischnitzer M., Zbedat K., Hochner-Celnikier D., Lavy Y., Yagel S. (2015). Degree of bother from pelvic floor dysfunction in women one year after first delivery. Eur. J. Obstet. Gynecol. Reprod. Biol..

[26] Volløyhaug I., Mørkved S., Salvesen Ø., Salvesen K.Å. (2015). Forceps delivery is associated with increased risk of pelvic organ prolapse and muscle trauma: A cross-sectional study 16-24 years after first delivery. Ultrasound Obstet. Gynecol..

[27] Friedman S., Blomquist J.L., Nugent J.M., McDermott K.C., Muñoz A., Handa V.L. (2012). Pelvic muscle strength after childbirth. Obstet. Gynecol..

[28] Dietz H.P., Shek K.L., Chantarasorn V., Langer S.E. (2012). Do women notice the effect of childbirth-related pelvic floor trauma?. Aust. N. Z. J. Obstet. Gynaecol..

[29] Dolan L.M., Hilton P. (2010). Obstetric risk factors and pelvic floor dysfunction 20 years after first delivery. Int. Urogynecol. J..

[30] Hage-Fransen M.A.H., Wiezer M., Otto A., Wieffer-Platvoet M.S., Slotman M.H., der Sanden M.W.G.N., Pool-Goudzwaard A.L. (2021). Pregnancy- and obstetric-related risk factors for urinary incontinence, fecal incontinence, or pelvic organ prolapse later in life: A systematic review and meta-analysis. Acta Obstet. Gynecol. Scand..

[31] Modroño Freire M.J., Sánchez Cougil M.J., Gayoso Diz P., Valero Paternain M., Blanco Ramos M., Cuña Ramos F.O. (2004). Estudio de prevalencia de incontinencia urinaria en mujeres de 18 a 65 años y su influencia en la calidad de vida [Study of the prevalence of urinary incontinence in women from 18 to 65 and its influence on their quality of life]. Aten Primaria.

[32] Johannessen H.H., Stafne S.N., Falk R.S., Stordahl A., Wibe A., Mørkved S. (2018). Prevalence and predictors of double incontinence 1 year after first delivery. Int. Urogynecol. J..

[33] Hosseini L., Iran-Pour E., Safarinejad M.R. (2012). Sexual function of primiparous women after elective cesarean section and normal vaginal delivery. Urol. J..

[34] Wang H., Ghoniem G. (2017). Postpartum stress urinary incontinence, is it related to vaginal delivery?. J. Matern. Fetal Neonatal Med..

[35] Blomquist J.L., Muñoz A., Carroll M., Handa V.L. (2018). Association of Delivery Mode with Pelvic Floor Disorders After Childbirth. JAMA.

[36] Schreiber Pedersen L., Lose G., Høybye M.T., Elsner S., Waldmann A., Rudnicki M. (2017). Prevalence of urinary incontinence among women and analysis of potential risk factors in Germany and Denmark. Acta Obstet. Gynecol. Scand..

[37] Barber M.D., Maher C. (2013). Epidemiology and outcome assessment of pelvic organ prolapse. Int. Urogynecol. J..

[38] Mankuta D., Shaul Y., Leshno M., Brezis M. (2007). 233: Spontaneous normal vaginal birth versus elective cesarean section by request—A decision analysis of maternal and perinatal complications. Am. J. Obstet. Gynecol..

[39] Gyhagen M., Bullarbo M., Nielsen T.F., Milsom I. (2013). A comparison of the long-term consequences of vaginal delivery versus caesarean section on the prevalence, severity and bothersomeness of urinary incontinence subtypes: A national cohort study in primiparous women. BJOG.

