# Supplementary material for: Pelvic Floor Morbidity Following Vaginal Delivery versus Cesarean Delivery: Systematic Review and Meta-Analysis
Source: J Clin Med. 2021 Apr 13;10(8):1652. doi: 10.3390/jcm10081652 (PMC8070303; doi:10.3390/jcm10081652)
Supplement: Supplementary file 1 [file jcm-10-01652-s001.pdf]

Annex 2

Publication risk of bias per disorder.

UI

Number of studies = 3 Root MSE = 0.6523

Std_Eff	Coef.	Std. Err.	t	$p > \|t\|$	[95% Conf. Interval]
slope	0.61339	0.100272	6.12	0.103	−0.6607–1.8875
bias	0.48164	0.766341	0.63	0.643	−9.2557–10.2189

Test of H0: no small-study effects $p = 0.643$
RESISTANCE FOR FUTURE NULL RESULTS (ROSENTHAL model)
The number of additional non-significant studies that are necessary to add a significant meta-analysis for becoming non-significant:73.
The number of unpublished studies 73 is >25 (k*5 + 10).

POP

Number of studies = 4 Root MSE = 1013

Std_Eff	Coef.	Std. Err.	t	p > |t|	[95% Conf. Interval]
slope	1.89124	0.353177	5.35	0.033	0.3716–3.4109
bias	−2.77292	1.319602	−2.10	0.170	−8.4508–2.9049

Test of H0: no small-study effects p = 0,170
RESISTANCE FOR FUTURE NULL RESULTS (ROSENTHAL model)
The number of additional non-significant studies that are necessary to add a significant meta-analysis for becoming non-significant:99.
The number of unpublished studies 99 is > 30 (k*5 + 10).

AI

Number of studies = 4 Root MSE = 1,618

Std_Eff	Coef.	Std. Err.	t	p > |t|	[95% Conf. Interval]
slope	0.52553	0.119651	4.39	0.048	0.0107–1.0403
bias	−1.34033	1.343679	−1.00	0,424	−7.1217–4.4410

Test of H0: no small-study effects p = 0.424

RESISTANCE FOR FUTURE NULL RESULTS (ROSENTHAL model)

The number of additional non-significant studies that are necessary to add a significant meta-analysis for becoming non-significant:65.

The number of unpublished studies 65 is >30 (k*5 + 10).
